# One-stage combined ENT and dental surgical treatment of odontogenic sinusitis: a prospective study

**DOI:** 10.1007/s00405-023-08332-y

**Published:** 2023-11-20

**Authors:** Petr Kocum, Jiří Šedý, Joseph Traboulsi, Petr Jirák

**Affiliations:** 1 Department of ENT, Head and Neck Surgery, Na Homolce Hospital, Roentgenova 37/2, Prague 5, Czech Republic; 2 Centre for Dental Surgery Podstata–Hudler, Ltd., Jinonická 1313/25, Prague 5, Czech Republic; 3 3DK Clinic, U Zdravotního Ústavu 2213/8, Prague 10, Czech Republic; 4 Faculty of Medicine, Institute of Dentistry and Oral Sciences, Palacký University, Palackého 12, Olomouc, Czech Republic; 5 Second Faculty of Medicine, Institute of Anatomy, Charles University, V Úvalu 84, Prague 5, Czech Republic

**Keywords:** Odontogenic sinusitis, Maxillary sinus, Prospective study, Human, Surgery

## Abstract

**Purpose:**

The study analyses outcomes of the surgical treatment of odontogenic sinusitis that concurrently address sinusitis and its dental source.

**Methods:**

A total of 364 adult patients were included, representing 13% of all patients we have operated on for any rhinosinusitis over the past 18 years. The diagnosis was based on both ENT and dental examinations including CT imaging. Patients were divided into three groups: (1) FESS with dental surgery without antrotomy, (2) FESS with intraoral antrotomy, and (3) intraoral surgery without FESS. The mean postoperative follow-up was 15 months.

**Results:**

First group involved 64%, second group 31%, and third group 6% of the cases. The one-stage combined ENT and dental approach was used in 94% of cases (group 1 and 2) with a success rate of 97%. Concerning FESS, maxillary sinus surgery with middle meatal antrostomy only was performed in 54% of patients. Oroantral communication flap closure was performed in 56% of patients (success rate 98%). Healing was achieved within 3 months. The majority (87%) of patients were operated on unilaterally for unilateral findings. Over the past 18 years, a 6% increase of implant-related odontogenic sinusitis was observed.

**Conclusion:**

Odontogenic sinusitis is common, tending to be unilateral and chronic. Its dental source needs to be uncovered and treated and should not be underestimated. Close cooperation between ENT and dental specialists has a crucial role in achieving optimal outcomes. The one-stage combined surgical approach proves to be a reliable, safe, fast and effective treatment.

**Supplementary Information:**

The online version contains supplementary material available at 10.1007/s00405-023-08332-y.

## Introduction

Odontogenic sinusitis (ODS), also known as dentogenic sinusitis or sinonasal complications of dental disease and treatment (SCDDT), accounts for up to 25–40% of all chronic maxillary sinusitis cases [1, 2].

Clinical cases of sinusitis fall into two nosologic categories [3, 4]. Briefly, in odontogenic maxillary sinusitis, the source is a dental pathology and the infection spreads “upwards”, whereas in rhinogenic maxillary sinusitis or rhinosinusitis, the source is in the nasal cavity or ostiomeatal unit (OMU) and spreads “downwards”. Odontogenic sinusitis has received less attention than other forms of sinusitis, despite its high prevalence [1]. In EPOS 2020 [5], according to the classification of secondary chronic rhinosinusitis, due to anatomic distribution (unilaterally localised) and endotype dominance (local pathology), odontogenic chronic rhinosinusitis is lumped together with fungal ball and tumours. There is no other tangible information on ODS in EPOS. Symptoms can differ (e.g., liquid leakage from the mouth to the nose is not considered a symptom of rhinosinusitis according to EPOS) and clinical findings and CT scan findings can also differ (endoscopic OMU findings can be subtle or none; on CT, in contrast to rhinogenic sinusitis, minimal thickening, involving only 1 or 2 walls and not the ostial area might represent odontogenic sinusitis).

Recently, cooperative efforts of experts in the field have led to the development of guidelines for ODS management [6]. In 2013, Felisati and Chiapasco introduced a classification of the so-called SCDDT and corresponding surgical therapy protocols [7]. This new concept was reacting to the boom in dental implantology which served as a source of additional complications and uncovered often misdiagnosed or underdiagnosed sinusitis of dental aetiology, mainly due to implementation of sinus lift (maxillary sinus floor bone augmentation procedure enabling the placement of dental implants in posterior maxilla). Another classification was formulated in 2020 [8], aiming at describing different possible combinations depending on the presence of sinonasal pathology and dental pathology or dental treatment. According to the international multidisciplinary consensus statement of 2021 [6], ODS refers to bacterial maxillary sinusitis, with or without extension to the other paranasal sinuses, secondary to either adjacent infectious maxillary dental pathology, or following complications from dental procedures. It is distinct from rhinosinusitis with no primary sinonasal inflammation.

Treatment of ODS usually requires therapy of both the sinusitis and its dental source, based on cooperation between an ENT specialist and a dentist. Surgical treatment involves endoscopic endonasal surgery and a range of dental surgical procedures and various modifications of intraoral approaches to the maxillary sinus. During these procedures, emphasis is placed on the complete removal of the cause of inflammation, preservation of the paranasal sinus function, and sparing of the alveolar process for future implant placement. The ideal variant of ODS remediation is to perform a combined surgery, i.e., Functional Endoscopic Sinus Surgery (FESS) and intraoral remediation at one time. Lopatin et al. [9] and Costa et al. [10] were the first and second researchers to report cases of ODS treated with a combined surgical approach. The first extensive study with 136 combined concurrent surgeries was published in 2013 by the Felisati group, which introduced the above-mentioned SCDDT classification [7].

Martu [11] stressed that treatment of ODS could be complex, usually involving both non-surgical and surgical stages; however, it is essential that the dental pathology is resolved; otherwise, an efficient, complete treatment is not possible. In the case of OMU involvement and previous oroantral fistula/dental implant with failed medical and dental therapy, the likelihood of the need for endoscopic endonasal surgery increases significantly [12]. Moreover, the oroantral communication (OAC) closure is much more likely to heal when restoring OMU patency [9, 13].

Our study aims to highlight the importance of a multidisciplinary ENT and dental approach to the ODS management and to emphasise the high success rate, reliability, and predictability of one-stage combined endoscopic (endonasal) and dental (intraoral) surgery. In some cases of ODS, there are reasonable reasons to prefer the conservative non-surgical treatment (e.g., endodontic and antibiotic), but we believe that in this disease, with all the logistical problems and factors which enter the decision process, in many cases, the combined and concurrent ENT and dental surgical approach can be the most effective method, and thus the most appreciated by the patient.

## Materials and methods

Our prospective study, examining three different surgical approaches in ODS, was designed according to Lopatin et al. [9] and modified according to our empirical experience, when a minimally invasive endonasal endoscopic technique combined with intraoral surgery was set to be superior to the Caldwell–Luc operation used for surgical treatment of chronic maxillary dentogenic sinusitis. This statement was later, during the course of the study, approved by other studies [7, 10, 14]. The study was approved by the institutional ethics committee of Na Homolce Hospital (03_F_NNH_064/16). All patients signed the informed consent.

Between 9/2004 and 1/2022, of a total of 2,791 adult patients operated on for rhinosinusitis in our department, 364 (13%) were treated for ODS concurrently addressing sinusitis and its dental source in all cases. The patients were divided into three groups, according to surgical approach aimed to solve the particular preoperative status/diagnosis:

GROUP 1 (“FESS with dental surgery but without intraoral antrotomy”; *N* = 231): patients treated by FESS with a dental surgery procedure (tooth extraction, implant extraction, periapical surgery and, if peroperatively developed, also oroantral communication closure) without intraoral antrotomy. The dental source was the tooth or implant indicated for extraction or the tooth after endodontic treatment indicated for periapical surgery.

GROUP 2 (“FESS with intraoral antrotomy”; *N* = 112): patients treated by FESS with intraoral antrotomy, cases technically possible to be solved by combined surgery with FESS. The dental source was oroantral fistula or odontogenic cyst, due to peri-implantitis infected sinus lift augmentation material, implant or tooth displaced in the maxillary sinus, microabscess in the fibrous tissue or bone sequestration in the maxillary sinus (with a Caldwell–Luc operation history).

GROUP 3 (“Intraoral surgery without FESS; *N* = 21): patients treated by intraoral sinus surgery without FESS, due to it being impossible to treat the state with the contribution of FESS (such as the tooth indicated for extraction being a source of infection to the fibrous tissue in the maxillary sinus in a patient with a Caldwell–Luc operation history with unrestorable function of the sinus).

We have defined ODS in our study as sinus or sinonasal disease in conjunction with dental pathology, while the following clinical aspects were considered in addition to the CT findings:

Symptoms: ranged from symptomatic rhinosinusitis (nasal obstruction, nasal discharge/foul smell, head and sinus pain/pressure, oroantronasal fluid penetration) to asymptomatic.

Intranasal endoscopic findings: varied from expressed findings with swollen mucosa, polyps, discharge, and lateral nasal wall medialisation with OMU obstruction to normal.

Dental disease in conjunction with sinus abnormalities was manifested as: (1) oroantral fistula, (2) dental pathology, which could (after its removal) cause oroantral communication with a need to perform surgical closure, or (3) any dental pathology indicated by shared decision for surgical treatment (such as a tooth possible to be saved by endodontic treatment). If one factor (for instance the tooth) was found multiply in one patient, even on both sides, it was counted only once, to be able to interpret the data.

Nasal/paranasal CT findings marking sinus pathology: (1) OMU opacification or (2) OMU patency with circular mucosal thickness in the maxillary sinus of 4 mm or higher [15–19].

Prior to surgery, all patients were examined by both an ENT specialist and a dentist. A standard examination protocol was used, including endoscopic examination of the nasal cavities and OMU (using a flexible endoscope) and dental examination of the teeth, accompanied by panoramic X-ray. The indication to perform CT scan was either symptomatic patient or intranasal clinical findings or previous finding of hyperplastic mucosa on panoramic X-ray/CBCT. CT of the paranasal sinuses was performed including the alveolar process of the maxilla with teeth/implants. In relevant cases, intraoral X-ray and/or cone-beam CT (CBCT) scans were performed. The criteria for dental aetiology were assessed according to possible dental causes: periapical/periodontal/combined inflammatory dental pathology (at time of treatment impossible to treat conservatively; dental provider or patient´s reason), peri-implantitis, odontogenic cyst, oroantral fistula, or foreign body in the paranasal sinus (tooth, radix, dental implant, and bone sequestrum). After the examinations, ENT made the indication for endonasal part of the surgery, whereas the dental surgeon for intraoral part of the surgery and, finally, the decision was shared by patient.

Surgery: it was performed concurrently by two specialists, ENT and dental; or by one specialist, ENT and dental in one person, in a one-stage procedure starting with the intranasal section, followed by the intraoral. Antibiotic prophylaxis was introduced intravenously 30 min prior to the surgery. If oroantral communication occurred and flap closure was performed and/or in case of finding pus in the maxillary sinus, the antibiotic use was prolonged to 7 days.

FESS: in patients where FESS was performed (groups 1, 2), surgical treatment was carried out to the extent according to the clinical findings and CT scan (1) at time of initial visit and (2) clinical findings immediately before and during surgery. At minimum, supraturbinal antrostomy (middle meatal antrostomy) and maxillary sinus treatment was performed. In the case of more extensive findings, anterior and medium ethmoidectomy and nasofrontal ostium treatment were carried out, always respecting the surgical conservatism rule. Sometimes, during the intranasal approach, supra- and infraturbinal antrostomy were combined. If the deviation of the nasal septum was significant for the ostiomeatal unit patency, septoplasty or partial septoplasty such as cristotomy or spinotomy was added. If the lower turbinate was hypertrophic, radiofrequency turbinoplasty or mucotomy was added.

Intraoral surgical approaches: intraoral surgeries included: extraction of tooth, root or dental implant together with surrounding inflammatory process removal (if the oroantral communication appeared peroperatively, it was followed by flap closure) (group 1); apical root resection (apicoectomy) with occasional retrograde root canal filling (group 1); excision of oroantral fistula with subsequent flap closure (group 2); removal of (due to peri-implantitis) inflamed sinus lift augmentation material (group 2); removal of implant/tooth previously displaced into maxillary sinus (group 2); modified Caldwell–Luc operation (groups 2, 3); extirpation of odontogenic cyst (groups 2, 3); treatment of post-Caldwell–Luc abscess or bone sequestration in maxillary sinus (groups 2, 3); classic Caldwell–Luc operation (group 3). A Rehrmann flap [20] was used more often than a palatal flap in oroantral communication closure. The modified Caldwell–Luc operation was intraoral minimally invasive maxillary sinus surgery, respecting the physiology of the paranasal sinuses, often performed alongside oroantral fistula excision, with the treatment of dental pathology displaced to the maxillary sinus, combined with subsequent OAC closure.

Inclusion criteria: complete medical documentation; verified diagnosis of odontogenic sinusitis, by each of the following: ENT examination, dental examination, CT of paranasal sinuses, panoramic and intraoral X-ray, nasal endoscopic examination; ability to follow up the patient for at least 2 months. Exclusion criteria: inability to obtain complete documentation; other diagnosis of sinusitis than odontogenic, e.g., rhinogenic sinusitis or tumour-related sinusitis; inability to follow up the patient for at least 2 months; patients operated on with FESS without solving the dental problem; patients operated on with FESS with dental problem already solved, e.g. after endodontic treatment; patients with dental problem (e.g., foreign body in sinus, odontogenic cyst) but without sinusitis; medication-related osteonecrosis of the jaw; patients with fibrous dysplasia of the maxilla or zygomatic bone.

In our study, augmented bone material infected by peri-implantitis was included in one of the dominant implant-related aetiology factors which was tracked: peri-implantitis, implant in maxillary sinus, oroantral fistula.

A range of parameters were studied (see the section “Results”).

Postoperative care followed standard protocol in our department.

Follow-up: Patients were followed up as long as possible (minimum 2 months). Mean follow-up after the surgeries was 15.8 ± 1.2 months (range 2–24 months). During follow-up, we updated the medical history and performed ENT and dental examinations as preoperatively. No X-ray examination was performed in successfully treated patients; the endonasal findings were verified endoscopically. CT scan and panoramic X-ray were indicated for the not-successfully treated, where the revision surgery was planned.

Successfully treated patients were clinically free of sinusitis symptoms (namely nose obstruction, nasal discharge, and sinus pain/pressure), with an endoscopically verified healed and functional ostiomeatal unit and healthy sinus mucosa, and had a completely healed alveolar process in the operated area, i.e., healed intraoral findings.

Intraorally incompletely (not-successfully) treated patients had one or more of the following: intraoral findings of unhealed maxillary alveolar process with dehiscence of the vestibular or palatal flap after OAC closure, oroantral fistula, unhealed extraction hole, signs of inflammation, e.g., swelling of the flap, discharge to the oral cavity, persistent pain.

Endonasally incompletely (not-successfully) treated patients had one or more of the following: endoscopically verified unhealed and/or non-functional ostiomeatal unit and sinus signs of pathology such as closed middle meatal antrostomy, persistent or enlarged mucosal hyperplasia in maxillary sinus or other sinuses, persistent pathological secretion from the sinus.

Combined intraorally and endonasally incompletely (not-successfully) treated patients had a combination of intraorally and endonasally unsuccessfully treated findings.

Data are reported as mean ± S.E.M. or percentage of total. Differences between groups were analysed using non-parametric Student’s *T* test using MS Excel with *p* < 0.001.

## Results

**Fig. 1:**
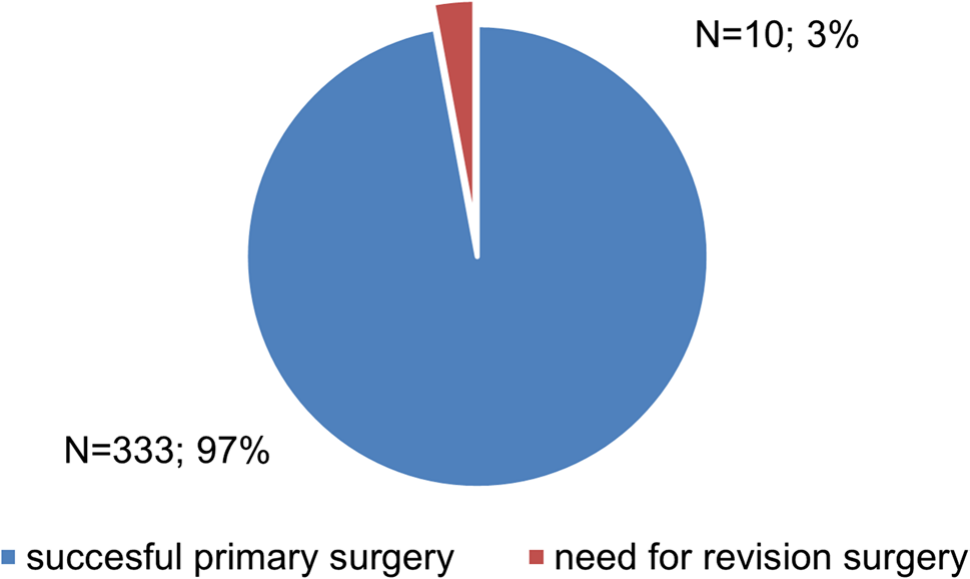
Success rate of the combined approach with FESS

Out of 364 patients, 224 (62%) were male and 140 (38%) were female. The mean age of the patients was 53.4 ± 19.5 years (range 21–82 years). The referring doctor was an ENT specialist in 226 (62%) patients and a dentist in 138 (38%) patients. Out of 364 patients, 354 (97%) patients suffered from chronic sinusitis and 10 (3%) patients had acute sinusitis. The majority of patients suffered from pain or pressure in the paranasal sinuses and/or head and experienced a nasal discharge and/or foul smell. Conversely, up to 8% of patients experienced no symptoms (Table 1). A single symptom was the presenting complaint in 173 (48%) patients. Such patients typically had pain or pressure in the paranasal sinuses and/or head, followed by nasal discharge/foul smell (Table 2). If we consider the EPOS RS definition [5], most of the patients had one or two symptoms (Table 3). The majority of patients had one symptom, fewer had two symptoms and the least had three symptoms or no symptom (Table 3). The symptom of leakage from the mouth to nose has been evaluated separately (Table 2); it was not included among the symptoms as requested by the EPOS definition. Concurrence of nasal obstruction and nasal discharge/foul smell (the two main symptoms of chronic rhinosinusitis according to EPOS) at the same time were present in 64 (18%) patients; nasal discharge/foul smell was observed more often than nasal obstruction (Table 1). Close to one-tenth of patients (29 of 364) had no symptom; most (17 out of 29) of those were cardiac patients examined and then operated on to eliminate the dental and ENT inflammatory focus before heart valve surgery. The medical histories revealed that allergy and smoking were common (Table 4). The combination of allergy and bronchial asthma occurred in 33 (9%) patients. The combination of chronic rhinosinusitis and paranasal sinus surgery was frequent. The combination of three history factors (chronic rhinosinusitis, paranasal sinus surgery, and allergy) was observed in 15 (4%) patients. The occurrence of particular aetiological factors is summarised in Table 5. In the vast majority of patients, the ODS was caused by a periapical/periodontal/combined process (Table 5). One aetiological factor was found in 318 (87%) patients, two in 41 (11%) patients and three in 5 (1%) patients. When analysing tooth vs. implant/augmentation procedure as an aetiology of ODS (total 373 aetiologies; bone sequestrum in sinus/Caldwell–Luc operation history were excluded), a tooth was found to be an aetiological factor in 325 (87%) cases and an implant and/or augmentation procedure in 48 (13%) cases. Out of 53 oroantral fistulae, 41 (77%) were of tooth origin and 12 (23%) were of implant origin. The patients were operated on by two individual specialists, ENT and dentist, in 130 (36%) cases; one surgeon combing two specialities (ENT and dentistry) only was involved in 234 (64%) cases. In the vast majority of cases (*N* = 343; 94%), the surgery was performed with a combined approach with FESS (Table 6). The FESS with dental surgery but without intraoral antrotomy (Group 1) approach was performed in 231 (64%) surgeries. Concerning the extent of FESS surgery, only maxillary sinus surgery with antrostomy was performed in 195 (54%) patients, while endonasal surgery to a larger extent was performed in 169 (46%) patients. Septoplasty or partial septoplasty was part of the surgery in 135 (37%) patients. OAC flap closure was performed in 205 (56%) patients; of these, 152 (66%) cases were operated in group 1 and 53 (47%) after oroantral fistula removals in group 2. All patients with unilateral findings were operated on the particular side only, whereas those with bilateral findings were operated on bilaterally. The right side was operated on in 143 (39%) patients, the left side in 173 (48%) patients, and both left and right in 48 (13%) patients. Thus, significantly more patients (*N* = 316; 87%) were operated on one side than bilaterally (*N* = 48; 13%) (*p* < 0.001). The mucosal (soft tissue) findings in the maxillary sinus during surgery are summarised in Table 7. The most common finding was mucosal hyperplasia or polyp. There was most frequently one finding (353 patients; 97%), two findings in 11 (3%) patients and three findings in 1 (0.3%) patient. The combination of both microabscess in fibrous tissue and mucocele was presented in 5 (1%) patients. The incidence of complications of surgery was low. Severe bleeding during surgery or within 1 day after surgery was presented in 4 (1%) patients. In one (0.3%) patient, the orbital plate of the ethmoid bone was fractured during FESS with a periorbital haematoma occurrence after the operation, with no postoperative visual disturbance or other possible harm. Postoperative nasal cavity adhesions were present in 23 (6%) patients. We did not observe any other severe complication, such as intraorbital or intracerebral bleeding, inflammatory complications of the surrounding soft tissues, orbital infections, brain abscess, cerebrospinal fluid fistula, meningitis, or any possible consequences of the above-mentioned. Patients spent mean 2.5 ± 1.2 days (range 2–5 days) in hospital. Importantly, 156 (43%) patients spent only 2 days in hospital; 65 (18%) patients 3 days and 14 (4%) patients spent 1 day in hospital. The mean follow-up was 15.8 ± 1.2 months (range 2–24 months). Healed findings were present up to 3 months after the operation, and no patient (of those followed up) had healing found present after this period. Healed nasal endoscopy findings were presented in 348 (96%) patients and healed intraoral findings were presented in 359 (99%) patients. Revision surgery due to unhealed findings (impossible to treat conservatively) was performed in 3% of patients (Table 8). In one of the revision modified Caldwell–Luc surgeries, due to oroantral fistula, the OAC re-closure was performed concurrently. The success rate of OAC flap closure in our study was 98% (5 reclosures after 205 closures). The success rate of the primary surgery performed in a combined approach with FESS (group 1 and 2) was 97%; 333 out of 343 patients (Fig. 1).

**Table 1:** Symptoms of patients treated for ODS (total 364 patients)

Nasal obstruction	Nasal discharge/foul smell	Pain/pressure in sinuses/head	Liquid leakage from mouth to nose	No symptoms
145 (40%)	187 (51%)	210 (58%)	29 (8%)	29 (8%)

**Table 2:** The only symptom of patients treated for ODS (total 364 patients)

Nasal obstruction	Nasal discharge/foul smell	Pain/pressure in sinuses/head	Liquid leakage from mouth to nose	No symptoms
27 (7%)	41 (11%)	71 (20%)	5 (2%)	29 (8%)

**Table 3:** Patients treated for ODS with/without symptoms of rhinosinusitis defined by EPOS

No symptoms	One symptom (main EPOS symptom or pain/pressure)	Two symptoms (at least one main symptom)	Three symptoms
29 (8%)	143 (39%)	126 (35%)	33 (9%)

**Table 4:** Data from medical history of patients treated for ODS (total 364 patients)

Allergy	Smoking	Chronic rhinosinusitis	Prior to heart valve surgery	Sinus surgery	Diabetes mellitus	Bronchial asthma
122 (34%)	113 (31%)	75 (21%)	59 (16%)	45 (12%)	41 (11%)	32 (9%)

**Table 5:** Aetiological factors of ODS (total 364 patients)

Periapical/ periodontal/ combined process	Oroantral fistula	Odontogenic cyst	Bone sequestrum in sinus/Caldwell–Luc operation history	Peri-implantitis	Implant in sinus	Tooth/root in sinus	Augmentation material
233 (64%)	53 (15%)	45 (12%)	25 (7%)	24 (7%)	12 (3%)	6 (2%)	0 (0%)

**Table 6:** Type of surgery performed for ODS (total 364 surgeries)

GROUP 1	GROUP 2	GROUP 3—MCL	GROUP 3—CL	Total
231 (64%)	112 (31%)	20 (6%)	1 (0.3%)	364 (100%)

*MCL* Modified Caldwell–Luc operation, *CL* Caldwell–Luc operation

**Table 7:** Soft-tissue surgical findings at maxillary sinus in patients operated for ODS (364 patients/surgeries)

Mucosal hyperplasia/polyp	Mucosal hyperplasia + pus	Mucosal hyperplasia + fungus ball	Retention cyst	Mucocele of sinus	Odontogenic cyst	Microabscess in fibrous tissue
214 (59%)	66 (18%)	28 (8%)	28 (8%)	11 (3%)	16 (4%)	15 (4%)

**Table 8:** Revision surgery performed after surgery for ODS (total 364 patients)

Revision surgery	Re-FESS	MCL	CL	OAC re-closure	Total
After all surgeries (GROUP 1, 2, 3)	5	2	1	4	12 (3%)
After surgeries with FESS (GROUP 1, 2)	5	1	0	4	10 (3%)

*MCL* Modified Caldwell–Luc operation, *CL* Caldwell–Luc operation, *OAC* oroantral communication

## Discussion

### One-stage combined and concurrent ENT and dental surgery for ODS

In ODS, it might be difficult to decide whether to use conservative or surgical treatment. Many factors need to be considered, such as the contribution of dental and rhinogenic pathologies including difficulties in detecting the dental origin, conflicts arising from involvement of two specialities including conflicts of public and private health providers, etc. However, cooperation is inevitable. For optimal outcomes of ODS treatment, it is necessary to accept the dental origin and necessity of patent OMU. Without solving the dental aetiology, there is no complete cure of ODS; and without the ENT contribution, the results are unpredictable and unreliable, with increased risk of severe sinusitis complications. In our study of 364 cases of ODS, two-thirds (64%) were operated on by a single ENT/dental surgeon, making the study more reliable by lessening the burden of interpersonal variability. The great majority of surgeries were performed in a one-stage combined ENT and dental approach with FESS. Importantly, the FESS with simple dental surgery but without intraoral antrotomy (group 1) was performed in two-thirds of surgeries. Undoubtedly, our study proves the very high success rate of the combined approach, which reached 97%. Several studies have shown similar results [7, 9, 10, 21–24], including a prospective study published by Saibene et al. [3], which validated the Felisati concept of SCDDT and its treatment protocol. With enlarging the sample size, our study encourages the proposed usage of a combined approach in the surgical treatment of odontogenic sinusitis. We observed healed findings intranasally and intraorally up to three months after surgery. According to our results, all ODS patients should be expected to be healed completely within 3 months, spending mostly 2–3 days in hospital, staying home for 1–2 weeks after surgery and using antibiotics orally for up to 1 week postoperatively. The surgical results are satisfactory and predictable.

Only in ten cases of the combined surgical approach did we have to perform revision surgery. The FESS part (five cases) failed due to a surgical oversight—closure of supraturbinal antrostomy due to rather small opening while undertaking a “small hole” antrostomy; the reason also might have been interference of concurrent rhinogenic disease (either chronic or acute). The intraoral part (four cases) failed in one case due to a large bone defect after large odontogenic cyst removal and in three cases due to a larger bone defect after removal of an implant and infected augmented sinus lift material with buccal flap closure. To re-close the oroantral fistulae, palatal flaps were used. Nine of the ten revision surgeries were successful with full recovery of the patients, while one patient (with a medical history of radiotherapy, MRSA positivity, and heavy smoking) remained “combined intraorally and endonasally incompletely treated”. Generally, one must distinguish between inaccurately performed surgery and recurrence of the disease due to persistent dental pathology (due to inaccurate diagnosis; or leaving pathology unsolved in the case of mixed dental pathology).

### FESS in ODS surgery

Thickened mucosa over 3 mm may be significantly associated with pathological findings in the posterior maxillary teeth [16]. Patients with 4 mm circular hyperplastic mucosa of the maxillary sinus on CT might be considered to be at higher risk of primary ostium dysfunction [15, 17]. Moreover, even with a patent ostiomeatal unit, oroantral communication closure without middle meatal antrostomy might be at higher risk of failure to heal. The value of FESS in speeding the recovery in oroantral fistula-related sinusitis has been recognised and proved [9, 13]. We have made similar observations. Currently, FESS can be considered the first-line therapy for symptomatic ODS, followed by dental treatment when necessary [25].

We prefer to perform FESS for ODS (1) when it is not feasible to treat the dental pathology conservatively (usually after repeated attempts of unsuccessful ENT and dental conservative treatment) or (2) when the responsible tooth or implant must be extracted with the risk of OAC creation, both while finding OMU obturation or hyperplastic mucosa in the maxillary sinus over 4 mm circularly or larger extent of paranasal sinus impairment on CT scan; (3) if there is a foreign body or mycetoma (fungus ball) to be removed from the maxillary sinus; or (4) if there is an OMU anatomical situation that can cause dysfunction of the maxillary sinus when solving the ODS prior to the intended sinus lift or its reoperation procedure.

### FESS extent in ODS surgery

In our study, in nearly half of the cases, FESS was performed to a larger extent than middle meatal antrostomy and maxillary sinus repair only, meaning performing ethmoidectomy and sometimes frontal sinus opening as well. The principle of middle meatal antrostomy only surgery, based on the “self-limiting” theory of extension to the anterior ethmoids and frontal sinus in odontogenic sinusitis [22, 26], might be generally reasonable; nevertheless, in our view, there is a need for an intraoperative decision according to the clinical status. Due to CT and mainly clinical findings, if there is pus in the ethmoids and frontal sinus or nasofrontal recess, if there is a risk of sinusitis complications, especially in an immunocompromised patient, we would recommend performing ethmoid opening and non-invasive frontal sinusotomy. The sufficient opening of the diseased sinuses resulting in the restoration of drainage and ventilation might be a matter of not only avoiding complications, but also faster resolution of the disease. The FESS complications per se in this situation are, as our study verifies, very improbable. The occurrence of dysfunctions or symptomatic synechiae in the nasal cavity was very low in our study. Even though a recent review [27] showed no clear predilection to sinusitis extent in odontogenic sinusitis-related orbital and intracranial complications, we are strongly convinced that odontogenic pus has no place in any sinus, especially close to the orbit and skull base. In all the above-discussed issues, more studies are necessary.

### FESS for ODS with oroantral communication or fistula

In two-thirds of group 1 cases in our study, OAC arose peroperatively after tooth or implant removal. Therefore, a dentist who performs removal of the tooth/implant with ongoing sinusitis should expect OAC often, and immediate flap closure without knowing the OMU status might have a high risk of failure to heal. As others have proved, the OAC closure is much more likely to heal with restoring OMU patency [9, 13]. The success rate of OAC flap closure in our study was 98%. Concerning the type of flap used for oroantral communication closure, in the majority of cases, we used a Rehrmann flap (buccal advancement flap), in the minority a palatal flap. In agreement with Molteni [28], we performed OAC closure no matter the size of the defect to maximise the chances of healing after a single procedure. Oroantral fistula (chronic epithelialised communication) must be excised and OAC closed.

### Surgery for dental implant-related ODS

Although the majority of sources of ODS are tooth-related, we observed a growing trend of origin from dental implant/bone augmentation procedures, being responsible for 13% of our cases. If we compared the years 2004–2012 and 2012–2022, there was a significant increase of implant-related aetiology from 8 to 14%. An even higher incidence (30%) was shown by the largest retrospective study containing 480 patients [28]. In our study, the by far most major aetiological factor in implant-related sinusitis was peri-implantitis. In our opinion, in that case, implant removal is fully indicated, as also suggested by others [29]. It should be understood that peri-implantitis as a cause of sinusitis, represents an unsolvable “Trojan horse” [29], and thus, without removal of the diseased implant, there is no full treatment of the disease. In infected displaced augmentation material following sinus lift, the combined surgical approach is a method of choice [7]; in our experience, in some cases where the mucoperiosteal flap is not capable of healing, FESS might be the method of choice, and as soon as the flap heals and is epithelialised, the intraoral approach is performed secondarily. This approach might be less invasive and so more appreciated by the patient. A dental implant was the cause of 23% of oroantral fistulae with sinusitis in our study. Importantly, the defect after implant extraction or loss, especially in inflamed sinus lift augmentation material, is usually large and with impaired vitality of the hard tissue, and leads to higher risk of OAC closure failure. To minimise this risk, it is recommended to use either double-layer closure [30], a palatal flap [31] or a Bichat fat pad [32]. Moreover, the major issue in dental implantology is currently the management of soft tissue (red aesthetics/function). An implant and crown surrounded by high-quality keratinised mucosa turned from the hard palate reduces the risk of peri-implantitis [33, 34]. An implantologist who was asked to reconstruct the dentition by inserting a dental implant into a position of a previously closed OAC would thus prefer a palatal flap [31, 34]. It is more difficult to perform, but the quality of the soft tissue on the top of the alveolar process (later forming the cuff around the implant and prosthetics) is much better.

The number of patients with combined ENT and dental surgeries of tooth-related sinusitis compared to implant-related sinusitis might differ in the future because of improving endodontic treatment and specific guidelines for when to prioritise conservative (endodontic) treatment over extraction of the tooth. As stated, we consider peri-implantitis with sinusitis an indication for implant removal. With the worldwide expansion of dental implantology, even though the osseointegration materials and the methods used by dentists are developing, complications are unavoidable. Nowadays, the role of dental implants in sinusitis must be understood and accepted and should not be underestimated. The proposed SCDDT treatment protocol [7] concerning the implant-related sinusitis meets our agreement. In our experience, it represents an instruction for a successful and effective treatment leaving patients with no residual inflammation process in the body. Its adoption by both ENT and dental specialists is warranted.

## Conclusion

Odontogenic sinusitis no longer represents a peripheral topic in rhinology. More than one-tenth of clinical and radiologic sinusitis has a primary dental pathology. Although tooth-related cases continue to represent the majority of ODS cases, we observed an increasing trend of sinusitis related to dental implants. Often, the disease is unilateral and chronic and could be asymptomatic; if symptoms are present, they may differ from those of rhinogenic sinusitis. The disease and its source should be properly treated and not be trivialised. The cooperation of ENT and dental specialists in solving odontogenic sinusitis is essential, and cooperative fusion of specialised expertise in the two fields is required for optimal outcomes. If both ENT and dental surgeries are indicated, the one-stage combined (concurrent) ENT and dental surgery represents a straightforward, reliable and curative approach, with low morbidity, low incidence of complications and generally rapid recovery, avoiding a series of unsuccessful multiple conservative (antibiotic) treatments and allowing for uncomplicated dental reconstructions.

### Supplementary Information

Below is the link to the electronic supplementary material.

Supplementary file1 (DOCX 62 KB)

## Data Availability

Not applicable.
