# Supplementary material for: One-stage combined ENT and dental surgical treatment of odontogenic sinusitis: a prospective study
Source: Eur Arch Otorhinolaryngol. 2023 Nov 20;281(3):1347–56. doi: 10.1007/s00405-023-08332-y (PMC10858141; doi:10.1007/s00405-023-08332-y)
Supplement: Supplementary file 1 — Supplementary file1 (DOCX 62 KB) [file 405_2023_8332_MOESM1_ESM.docx]

**Supplementary material**

**Terms used in the study:**

**CBCT** – cone beam CT

**CL** – Classic Caldwell-Luc operation: an intraoral approach to the maxillary sinus, using the anterolateral access with bony window creation, sinus mucosa removal and creation of bony window to the inferior nasal meatus, with no subsequent window restoration.

**CRS –** chronic rhinosinusitis

**CT** – computed tomography

**DS –** dentogenic sinusitis

**EES** – Endoscopic Endonasal Surgery

**EPOS 2020 (EPOS) –** European Position Paper on Rhinosinusitis and Nasal Polyps 2020 [1]

**EX** – Extraction/Explantation: removal of tooth/root/dental implant from the alveolar process.

**FESS** – Functional Endoscopic Sinus Surgery: a minimally invasive set of techniques used to restore ventilation and normal function of the paranasal sinuses. In SCDDT with sinusitis, the following intranasal procedures are used: OM unit treatment, supraturbinal antrostomy (middle meatus antrostomy), infraturbinal antrostomy (endoscopic transnasal anterior approach to the maxillary sinus) [2], ethmoidectomy, surgical approach to the frontal recess and frontal sinus.

**FESS+EX/PAS; FESS+MCL – Combined (multidisciplinary, concurrent) approach**: the endoscopic and intraoral approach combined, in one-stage surgery.

**IMCS** – International Multidisciplinary Consensus Statement on diagnosing odontogenic sinusitis, Craig, Poetker, Aksoy, et al. 2021 [3].

**MCL** – Modified C-L surgery: a minimally invasive modification of the C-L operation. It fully respects the physiology of the paranasal sinuses with OMU as a crucial region and the need for mucosa preservation. It is often performed together with OAF excision, with the treatment of dental pathology / foreign body displaced to the maxillary sinus, combined with subsequent OAC closure. It is often combined with FESS. It enables revision of the alveolar recess of the maxillary sinus (also with endoscopic instruments), which is difficult to reach via FESS. It includes bone-lid procedures.

**MMA** – Middle Meatal Antrostomy, i.e. Supraturbinal Antrostomy

**OAC closure** – Oroantral communication closure: an intraoral surgical procedure using local mucoperiosteal flaps to close the communication between the oral cavity and maxillary sinus. A buccal advancement flap (Rehrmann flap / Wassmund flap), Palatal flap or Fat pad flap (Bichat flap) is usually used.

**OAF excision and closure** – Oroantral fistula removal and closure: represents complete remediation of the fistula. It consists of OAF excision and subsequent OAC closure.

**OC extirpation** – Odontogenic cyst extirpation: complete removal of the cyst. If the cyst is too large and the bone pushed out into the maxillary sinus by its expansion is infected and/or unstable, the so-called Neoantrum is surgically restored, meaning complete removal of not only the cyst but the adjacent bony shell as well.

**ODS** – odontogenic sinusitis

**OM unit (OMU; OMC, ostiomeatal complex)** – ostiomeatal unit or ostiomeatal complex: a common channel that links the [frontal sinus](https://radiopaedia.org/articles/frontal-sinus?lang=us), anterior [ethmoid air cells](https://radiopaedia.org/articles/ethmoidal-air-cells?lang=us) and the [maxillary sinus](https://radiopaedia.org/articles/maxillary-sinus?lang=us) to the [middle meatus](https://radiopaedia.org/articles/middle-meatus?lang=us), allowing airflow and mucociliary drainage. It is a functional region that includes the uncinate process, ethmoidal infundibulum, anterior ethmoid cells with their ostia, and the ostia of the maxillary and frontal sinuses. An obstruction at this level can be the trigger for disease, potentially involving the whole anterior compartment (frontal sinus, maxillary sinus, anterior ethmoid) [2]. It is a crucial region for SCDDT sinusitis.

**Osteoplastic intraoral approach** **(bone-lid procedure)** – an intraoral approach with creation of a bony window pedicled to Schneider´s membrane [2]. It is made on the anterolateral maxillary sinus wall and works as a door which is opened and closed and fixed with resorbable sutures. It enables foreign body removal with preservation of the anterolateral sinus wall. It is appreciated by dental implantologists, when later performing lateral sinus lift procedure (see term below).

**PAS** – Periapical surgery: a revision of the periapical region of a tooth, removing inflammatory process or cyst after endodontic treatment. It sometimes includes Apical resection / apicoectomy (removal of the root tip of the tooth) and/or retrograde MTA filling.

**Peri-implantitis –** an inflammatory process affecting the tissue around an implant that results in loss of supporting bone [4].

**PNS** – paranasal sinuses

**RS** – rhinosinusitis

**SCDDT –** sinonasal complications of dental disease and treatment, Felisati/Chiapasco classification

**SCDDT** **associated with sinusitis** – the only SCDDTs where the sinusitis/rhinosinusitis is expressed (Felisati/Chiapasco groups I, II, III except class 2d (implant displacement without sinusitis)); see definition above

**SL** – sinus lift; maxillary sinus floor augmentation: a [surgical procedure](https://en.wikipedia.org/wiki/Surgery) which aims to increase the amount of bone in the [posterior](https://en.wikipedia.org/wiki/Commonly_used_terms_of_relationship_and_comparison_in_dentistry) [maxilla](https://en.wikipedia.org/wiki/Maxilla) by lifting the lower [Schneiderian membrane](https://en.wikipedia.org/wiki/Schneiderian_membrane) (sinus membrane) and placing a bone graft; it is used for dental implant placement in some cases of vertical bone insufficiency.

**SP** – Septoplasty: surgical correction of a septal deviation or septal ridge/spur removal (spinotomy/spinectomy, cristotomy/cristectomy). Can be performed in a traditional or endoscopic way. In SCDDT sinusitis, it is used for remediation of middle meatus (OMU) function or for approach purposes (removal of obstacle for the instruments) [2].

**TP** – Turbinoplasty of a lower turbinate: performed to reduce the volume of the turbinate and restore the nasal cavity ventilation.

**TP RF** – radiofrequency turbinoplasty: in some cases used in our patients as part of FESS.

**METHODS SUPPLEMENT**

**Surgery**

Surgery was performed by an ENT surgeon and dental surgeon in a one-stage procedure, starting with the intranasal section, followed by the intraoral. The patients were operated on concurrently by two specialists, ENT and dental; or by one specialist, ENT and dental in one person. The operating team consisted of two surgeons and a nurse. Premedication (Alprazolamum 0.25–0.5 mg; Frontin 0.25 mg, Egis Pharmaceuticals PLC, Hungary) was introduced orally 60 minutes prior to the surgery. Antibiotic prophylaxis Amoxicillinum 1000 mg with Acidum clavulanicum 200 mg (Amoksiklav 1.2g, Sandoz Novartis, Switzerland) in a 1.2 g dose accompanied by Ampicillinum 1000 mg (Ampicilin BBP 1g, BB Pharma, Czech Republic) in a 1–3 g dose according to the weight of the patient was introduced intravenously 30 minutes prior to the surgery. In the case of allergy to penicillin antibiotics, Clindamycinum (Clindamycin Kabi 150 mg/ml, Fresenius Kabi, Czech Republic) in a 600 mg dose was introduced intravenously 30 minutes prior to the surgery. General anaesthesia was provided by an anaesthesiologist. Orotracheal intubation was performed, positioning the tube on the non-operating side of the oral cavity. The general anaesthesia was supplemented by local intranasal topical anaesthesia, Hirsch solution (Cocaini chlorati 2.0, Kalii sulfuric 0.5, Phenoli liquefacti 0.5, Sanorini 1‰ 10.0, Aqua purificata ad 100.0) administered on cotton tissues in the nasal cavities 10 minutes prior to the intranasal part of surgery; and infiltration intranasal anaesthesia, Trimecaini hydrochloridum (Mesocain 10mg/ml, Zentiva, Czech Republic) and Epinephrinum (Adrenalin Léčiva, 1mg/ml, Zentiva, Czech Republic), using 4 drops of Epinephrine in 10 ml of Mesocain. Intraorally articain anaesthesia (Supracain 4%, Zentiva, Czech Republic) was added. Both the intranasal and intraoral anaesthesia were applied according to the standard use in ENT and dentistry [5, 6]. During the intranasal part of the surgery, blood pressure of about 100/60 mmHg was required. Antibiotic prophylaxis was introduced intravenously 30 minutes prior to the surgery. If oroantral communication occurred and flap closure was performed and/or in the case of finding pus in the maxillary sinus, the antibiotics use was prolonged to 7 days or more according to microbiology specialist consultation of a peroperatively taken sample. Pre-operative administration of oral steroids was not used, although at the end of surgery, 200 mg intravenously of Hydrocortisoni hydrogenosuccinas (Hydrocortison VUAB 100mg, VUAB Pharma, Czech Republic) was used as a one-off.

**FESS**

In patients where FESS was performed (groups 1, 2), surgical treatment was carried out to the extent according to the clinical findings and CT scan (1) at the time of the initial visit and (2) clinical findings immediately before and during surgery. At minimum, supraturbinal antrostomy and maxillary sinus treatment were performed. In the case of more extensive findings, anterior and medium ethmoidectomy and nasofrontal ostium treatment were made, always respecting the surgical conservatism rule. The size of the supraturbinal antrostomy (usually around 10 mm) was determined by the intra-sinus findings [5]. If a foreign body or mycetoma had to be extracted, sometimes we were forced to approach the maxillary sinus infraturbinally (through the wall of the nasal cavity under the inferior turbinate), opening and closing the bony door pedicled to the nasal wall mucosa [7]. Sometimes, during the intranasal approach, supra- and infraturbinal antrostomy were combined. If the deviation of nasal septum was significant for the ostiomeatal unit patency, septoplasty or partial septoplasty such as cristotomy or spinotomy was added. If the lower turbinate was hypertrophic, turbinoplasty RF or mucotomy was added. At the end of surgery, an absorbable haemostatic gelatine sponge Spongostan (Ethicon, Johnson-Johnson surgical technologies, USA) was inserted into the middle meatus; in risky conditions (hypertension, LMWH of higher dose usage), a tamponade in rubber was embedded. If septoplasty was performed, a bilateral tamponade was always inserted.

**Intraoral procedures**

Intraoral surgeries included:

(1) extraction of tooth, root or dental implant together with surrounding inflammatory process removal; if the oroantral communication appeared peroperatively, it was followed by flap closure (group 1),

(2) apical root resection (apicoectomy) with occasional (if indicated) retrograde root canal filling with MTA reparative cement (MTA Angelus, Angelus, Brazil) (group 1);

(3) excision of oroantral fistula with subsequent flap closure (group 2),

(4) removal of (due to peri-implantitis) inflamed sinus lift augmentation material (group 2),

(5) removal of implant/tooth previously displaced into maxillary sinus (group 2);

(6) modified Caldwell-Luc operation (groups 2, 3),

(7) extirpation of odontogenic cyst (groups 2, 3),

(8) treatment of after CL operation abscess or bone sequestration in maxillary sinus (groups 2, 3);

(9) classic Caldwell-Luc operation (group 3).

When closing oroantral communication, Rehrmann flap closure [8] was performed in the majority of cases, less often a palatal flap was used. The suture was done with a non-absorbable monofil 5-0 material (Resolon 5-0, DS 18, Resorba, Germany), often using mattress stitches. Special attention was paid to tension-free closure.

Concerning the sinus lift, we differentiated sinusitis with infected sinus lift augmentation (augmented) material due to peri-implantitis (chronic scenario; SCDDT group II except class 2d) from sinusitis with infected displaced/not-displaced sinus lift augmentation material following maxillary sinus grafting (usually acute/subacute scenario – SCDDT group I). The latter case, the case of primary sinus lift failure, was excluded from the study. Those were either operated on with FESS and without solving the dental problem intraorally or were operated on with FESS with the dental problem already solved. In our study, augmented material infected by peri-implantitis was included in one of the dominant implant-related aetiology factors which was tracked: peri-implantitis, implant in maxillary sinus, oroantral fistula.

**Modified Caldwell-Luc** **surgery**

In this study, modified C-L surgery was defined as minimally invasive maxillary sinus surgery using an intraoral approach, respecting the physiology of the PNS. It was often performed with OAF excision, with the treatment of dental pathology displaced to the maxillary sinus, combined with subsequent OAC closure. Access to the maxillary sinus was made by a bony window, where the bone lid was either removed or preserved in situ (bone-lid procedure). This approach enabled the use of endoscopic instruments as needed as well. In some cases, it was convenient to only enlarge the post-extraction oroantral communication in order to use it as a mini-invasive access to the sinus. The mucosa was preserved as much as possible, removing only the pathology. This also enabled the revision of maxillary sinus alveolar recess, which is difficult to reach via FESS. In case of combined operation with FESS (group 2), drainage was secured either by supraturbinal antrostomy itself or by inserting the drain through the supraturbinal antrostomy for two days postoperatively (to enable rinsing of the sinus postoperatively). The suture was done with a non-absorbable monofil 5-0 material (Resolon 5-0, DS 18, Resorba, Germany), mostly using mattress stitches. In our study, temporary placement of a tubular drain infraturbinatelly from the maxillary sinus to inferior meatus of nasal cavity in group 3 was not considered to be a FESS/EES approach.

**Postoperative care**

Postoperative care followed the standard protocol in our department. After the surgery, patients were hospitalised for two days at the clinic and monitored. After FESS and septoplasty, if a nasal tamponade was used, it was removed 24 hours postoperatively. If Spongostan (Ethicon, Johnson-Johnson surgical technologies, USA) was inserted into the middle meatus, it was not removed until the first inpatient check one week postoperatively. After two nights of hospitalisation, the patient underwent simple cleaning of the lower nasal meatus on the operated side. Then, it was recommended to stay at home till the first check one week after, using decongestive nasal drops, analgesics and nose-rinsing with mineral water (Vincentka nasal spray 0.25 ml, Vincentka, Czech Republic). In addition, patients came for a check with cleaning of nasal and paranasal cavities once a week for two weeks and once every two weeks for at least two months postoperatively. Concerning the intraoral part of surgery, for the first two days external cooling of the cheek on the operated side was recommended, the patients did proper oral hygiene without any harm to soft tissue of the operated area, with no rinsing of the suture for four days. In the case of oroantral communication closure, specific instructions were given: patients were instructed not to blow their nose and if they needed to sneeze, to do so with an open mouth for 3 weeks, and not to smoke; they used antibiotics Amoxicillinum 875 mg with Acidum clavulanicum 125 mg (Amoksiklav 1 g, Lek Pharmaceuticals, Slovenia) orally at 1g every 8 hours for 7 days (in case of penicillin allergy, they used Clindamycinum (Dalacin C 300 mg, Pfizer, USA) orally at 300 mg every 8 hours for 7 days). If pus was present, the antibiotics were modified after microbiology specialist consultation as needed. Stitches were removed 2 weeks after surgery.

**Studied parameters**

We studied the following parameters: sex; age; referring specialist – ENT/dentist; clinical course – acute/chronic; symptoms – nasal obstruction, nasal discharge/foul smell, pain/pressure in PNS/head, oroantronasal fluid penetration/leakage; a reduction or loss of sense of smell was not evaluated; medical history; type of dental/dentogenic aetiology – periapical/periodontal/combined inflammatory dental pathology, oroantral fistula, odontogenic cyst, bone sequestration–CL operation history, peri-implantitis, foreign body in paranasal sinus–dental implant, tooth, root; surgeon – two specialists in one person / two specialists in cooperation; type of surgery – FESS+EX/PAS, FESS+MCL, MCL, CL; septoplasty as part of surgery, including partial septum endoscopic surgery; FESS surgery extent – maxillary sinus only / more than maxillary sinus; oroantral communication flap closure; side of pathological findings and surgery; surgical finding in maxillary sinus; complications of the surgery – bleeding, adhesions in nasal cavity; healing after surgery – endoscopic findings, intraoral findings; revision surgery; length of hospitalisation – days spent in hospital; length of follow-up.

**DISCUSSION SUPPLEMENT**

Sinusitis caused by dental disease or dental treatment is by its very nature an interdisciplinary issue. Thus, inherently, it should be treated as a cooperation between the two disciplines – ENT and dentistry. Although different designations (nosologic classes) have been given to the disease, whether we call it odontogenic sinusitis (ODS) or sinonasal complications of dental disease and treatment (SCDDT) or dentogenic sinusitis (DS), it still remains true that the disease has undoubtedly a dental cause and sinusitis as a consequence. The terms “odontogenic” and “dentogenic” can be used as synonyms, since the first originates in Latin and the latter in Greek (odūs, odontos; dens, dentis). In ODS, obviously, it would be best to treat the dental problem (especially conservatively) and solve the disease. Different combinations of conservative and surgical ENT and dental treatments might be considered. If surgical treatment is indicated, the concurrent approach of an ENT and dental surgeon is very convenient for all three participants – patient, dentist and ENT specialist. Very often though, it is a “logistical problem”, depending mainly on willingness to cooperate and to understand. What the patient needs is efficiency and time saving. A patient with odontogenic sinusitis usually comes after one or several antibiotic courses, having circulated among an ENT doctor, dental provider or GP (emergency units). Indeed, another factor is the general practitioner, who is often the first to, usually several times, treat the disease with antibiotics. Not only otolaryngologists and dental providers, but also GPs should have knowledge of ODS. All medical participants should bear in mind that more than one-tenth of sinusitis cases generally might be of dental origin. From a medical point of view, we consider the one-stage combined surgery the best choice for the patient, with all respect to the different factors that come into account in diagnosis and assessment of management of the disease. Decision-making should be shared [9]. Nevertheless, we believe that in some of the cases ENT should decide, in some the dental specialist should decide and in some the patient should decide.

**Incidence of the disease**

A total of 364 adult patients have been operated on for ODS, which is 13% of all patients operated on for the diagnosis of rhinosinusitis in our department in the monitored years. Some studies show [9, 10], that up to 25% to 40% of all chronic maxillary sinusitis cases might have dental origin. In reality, it is practically impossible to obtain the exact number, but there is a strong conviction that around 20%, every fifth patient, with maxillary sinusitis might have ODS (unpublished observation). Our department is not only specialised in rhinology, but we also focus on patients with nearly the whole spectrum of ENT diseases; on the other hand, patients with ODS are referred to our department for concurrent surgeries preferentially. This fact, which will be discussed hereafter, may limit the impact of the conclusions.

**Chronic disease, symptomatic disease, complications of the disease**

Many of the acute ODS patients come to ENT specialists first, because of the sinusitis symptoms. If the acute odontogenic sinusitis shows no complications of the disease (e.g. orbitocellulitis), there is no need to operate on the sinus at first. There is a crucial need to suspect dental origin, though. The ENT treatment is usually conservative, using antibiotics and sometimes maxillary sinus punctures with rinsing. When the treatment is not successful, with variable treatment length and after variable (usually multiple) antibiotic courses, the patient is referred to a dentist. The dentist finds or does not find the dental origin and either performs conservative or surgical treatment or excludes the dental origin. A proper precise dental examination is essential because the dental origin is sometimes difficult to find. The same precision must be used in the dental treatment. With these difficulties the process can be prolonged. After the proper treatment of the dental source and treatment of the sinus, ODS can be healed. If not, the patient presents with repeated relapse or with chronic disease. Chronic ODS patients come to either ENT or the dentist primarily, sometimes with bland symptoms or asymptomatic; the disease can be found within clinical examination and mainly on CT (CBCT). Sometimes the dental origin is more than obvious and is detectable even for the ENT specialist. Moreover, the later discussed features should lead the specialist to suspect ODS. There remains a large window for improvement of the diagnostic process – if the ENT specialist takes the odontogenic origin into account at the first line, there is much lower probability of failure of the first-line treatment [11]. The number of chronic diseases as well as the sinusitis complications would decrease. Not only acute sinusitis, but also chronic can lead to severe complications, such as orbitocellulitis, brain abscess or osseous complications [12]. The literature also suggests treating patients with both acute and chronic ODS, to avoid its complications [12].

**Symptoms, findings, unilaterality**

Our study shows that almost one-tenth of patients can have no symptoms. If there are symptoms, it will be more likely pain or pressure in PNS or head and rather nasal discharge (often with foul smell) than nasal obstruction when we examine the main EPOS RS symptoms. Moreover, one-third of patients were allergy sufferers, one-fifth had a history of chronic rhinosinusitis and in one-third of patients, septoplasty or partial septoplasty was a part of surgery. All these three factors can potentially lead to nasal obstruction, thus increasing the false positivity of a nasal obstruction symptom in our study. The ENT specialist must exclude tumours and consider the possibility of dental origin, especially in unilateral findings. Some studies have shown ODS accounting for 45% to 75% of unilateral maxillary sinus opacification on CT [13-17]. In our study, 87% of patients had unilateral findings and surgery. The unilaterality of the process should be the leading signal that should not be omitted. According to an international multidisciplinary consensus statement on diagnosing ODS (IMCS, Craig and colleagues, 2021) [3], and in agreement with the results of our study, the overwhelming majority of ODS is unilateral, so both ENT and dental providers should suspect dental origin in the setting of unilateral maxillary sinusitis.

**Endoscopic findings and CT in relation to diagnosis assessment; to later dental reconstruction**

The diagnosis assessment is crucial to deciding the proper management of the disease. We did not track the OMU endoscopic findings prior to surgery; later the OMU findings were reported as not exclusive for diagnosis [3]. However, we aimed at tracking them in the maxillary sinus during surgery. More than half of the findings were mucosal hyperplasia or polyps, in one-fifth of the cases accompanied by pus. Decision-making based on clinical findings at OMU only may be inconclusive or misleading, since endoscopic findings at OMU depend on at what stage (of the disease and also of the dental treatment) the patient comes to the ENT doctor and can vary from expressed findings with swollen mucosa, polyps, nasal discharge, lateral nasal wall medialisation with OMU obstruction to normal. In our view, at the time of treatment decision in ODS, imaging is more important than clinical findings at OMU. After the surgery, endoscopic postoperative checks can provide the same or better information about the functional status of the sinuses and reduce the need for imaging, thus decreasing the radiation burden on the patient. And in this context, postoperative endoscopy should be appreciated by the dental implantologist, who should understand that the function of the nose and sinuses is of high importance for dental implant reconstruction.

**CT and CBCT**

Nevertheless, it must be emphasised that every imaging modality has its limits. In agreement with Whyte et al. [18], in the case of performing CT (usually otolaryngologist), we suggest CT of PNS and the whole alveolar process of the upper jaw with teeth/implants; in the case of performing CBCT (usually dental provider), we suggest CBCT of the upper jaw with teeth/implants with OMU to be visualised; i.e. “down-visualisation” on CT and “up-visualisation” on CBCT. This extent of visualisation must not be omitted even in the case of a clinically edentulous jaw (radix with apical periodontitis, residual odontogenic cyst, infected implant or augmentation material in the alveolar process, etc.). This precaution should help in reducing misdiagnosis of odontogenic sinusitis. The latter also helps the implantologist to evaluate the OMU condition before performing SL [19]. In the case of FESS planning in ODS, we recommend that all PNS are visualised. Negative imaging of dental pathology with ongoing suspicion should be subject to advanced endodontic examination of the teeth and closer evaluation of the prosthetics (crowns but mainly bridges) in implants. History taking and correct information from the dental provider is important. Emphasis must be put on the timing of the imaging procedure. In our study, we did not treat each patient with antibiotics before the CT scan and decision-making and prior to surgery, which set different starting positions. False positivity in imaging PNS is possible due to the interval after the endodontic treatment, which would influence the decision concerning the FESS extent, if FESS is indicated. The interval should be studied and the extent of the FESS approach should be adjusted intraoperatively. Also, at the time of assessment of diagnosis of ODS, no acute rhinogenic rhinosinusitis should be present to avoid false positivity on CT. Fungus ball (FB) in the maxillary sinus is associated with dental procedures, especially endodontics [7]. In almost one-tenth of the cases, we found FB, often asymptomatic. Sometimes there was no dense opacification typical of FB on the CT scan, or the FB finding in the maxillary sinus during surgery was surprisingly much larger than expected from the CT scan.

**Suspecting ODS in relation to IMCS, EPOS**

According to an International multidisciplinary consensus statement (IMCS) on diagnosing odontogenic sinusitis [3], the features that can facilitate ODS suspicion are disease unilaterality, symptoms, nasal endoscopy findings, bacterial sinus cultures, and CT findings. The importance of unilateral finding was confirmed by our study. Regarding symptoms in our study, the most common single symptom was pain or pressure in the PNS and/or head, followed by nasal discharge. The discharge, in agreement with the IMCS, is usually accompanied by a foul smell. Concurrence of nasal obstruction and nasal discharge (two main symptoms of CRS according to the EPOS definition) at the same time were present in almost one-fifth of patients, when nasal discharge was observed more often than nasal obstruction. Regarding nasal endoscopy, as mentioned, we did not track the findings prior to surgery (at the time of diagnosis/management decision-making), although we did track them intraoperatively. Almost every fifth case had pus in the maxillary sinus. Regarding bacterial sinus cultures, patients used antibiotics as prophylaxis of surgery, the cultures were obtained from the maxillary sinus during surgery if pus was present, and in that case, the patients continued using antibiotics for 7 days after surgery or longer according to microbiology specialist consultation of a peroperatively taken sample (see Methods – Surgery and Postoperative care). Regarding CT imaging, in almost half of the cases the opacification was in an extra-maxillary extension. Empirically, we can confirm the spread to the anterior ethmoids and frontal sinus rather than to the posterior ethmoids and sphenoid sinus [16, 20-22]. We strongly support the need for dental examination if otolaryngologists find opacification in the maxillary sinus on CT. CT may not be sufficient for dental pathology detection and CBCT (or another diagnostic method) must be performed [3].

**Confirming sinusitis, confirming odontogenic sources of sinusitis in relation to IMCS**

According to IMCS [3], symptoms *per se* are not required for diagnosing ODS. We are in agreement with the statement: every tenth patient in our study was asymptomatic. Nasal endoscopy was considered the most important method for confirming sinusitis in ODS, with CT scan adding further support. The IMCS stated that nasal endoscopy confirms infectious ODS more reliably than CT, when isolated mucosal thickening in the maxillary sinus and mucus retention cysts on CT generally do not represent ODS, unless there is concurrent nasal endoscopic evidence of infection or inflammation. From our experience, mucosal thickening in the maxillary sinus (due to dental pathology) in imaging can represent both an infectious and a non-infectious situation. Tooth pathology and peri-implantitis are most often infectious processes [23, 24]; implant protrusion up to 2–3 mm into the maxillary sinus [25-27] will most probably invoke a non-infectious reaction of the mucosa to the foreign body with no need for treatment in most cases. Endoscopic findings at OMU depend on at what stage the patient comes to otolaryngologist and can vary from expressed findings to normal. Presumably, most of the one-tenth of asymptomatic ODS in our study had no discharge or other finding in OMU, but they had at least 4 mm of hyperplastic or mucosal thickening in the maxillary sinus with patent OMU and dental pathology on CT or found by dental examination. In our opinion, these patients should be treated as patients with ODS because of dental infection. There is no doubt that from a medical point of view, inflammation of dental origin generally must be treated, among other indications, as a source of focal infection [23]. Complications of dental infection with subtly expressed sinusitis (in terms of asymptomatic mucosal thickening) can arise in a haematogenous way, with no sinusitis suffering (brain abscess, endocarditis, etc.) [28, 29]. Although we agree that OMU findings may be a leading sign for the confirmation of sinusitis in ODS, in our view, at the time of the treatment decision in ODS, imaging together with referred history of symptoms may be more important than immediate clinical findings at OMU. Unfortunately, in our study we did not track the percentage of cases with positive endoscopy findings at OMU at the time of diagnosis, i.e. management decision-making. By definition, ODS is a bacterial disease, so sooner or later, purulent discharge will develop. But waiting for discharge in OMU, thus postponing the treatment, may lead to odontogenic sinusitis complications. In that matter, especially in the department where we perform surgeries on referred patients, we must rely on referring otolaryngologists as well. In medical history, we often find repeated nasal discharge. In our study two-thirds of referring specialists were ENT doctors. From that we deduce that not only ENT-dental but also inter-ENT cooperation is valuable. Thus, the crucial point for the proper diagnosis is the stage of the disease at which the patient is evaluated. In agreement with the IMCS [3] patient with negative nasal endoscopy but with CT findings and suspicious symptoms should proceed to a dental provider, and sinusitis in ODS can be tentatively confirmed. The decision about mucosal thickening (if a rhinogenic cause has been excluded), whether it is infectious or non-infectious, is then up to the dental provider. Concerning the detection of the dental pathology, the most often used pulp vitality tests and/or imaging methods such as CBCT might be inconclusive in some cases, so a more complex diagnostic process is necessary.

**Further comments on performed surgeries.**

FESS/EES is based in respect to the physiology of the PNS and so represents a minimally invasive approach. In ODS cases, the dental pathology mostly needs to be treated intraorally, enabling treatment of the dental source and reaches the alveolar recess and the ventromedial portion of the maxillary sinus, where the endonasal endoscopic approach meets its technical limits [30]. When operating intraorally, the endoscopic approach can be used as well [31] and sometimes a minimally invasive intraoral approach such as the bone-lid technique is also recommended [30, 32]. According to some reviews [19, 33], and to the author´s knowledge, there is no consensus regarding the cut-off point beyond which mucosal thickening of the maxillary sinus should be regarded as pathological: the distinction between healthy and diseased sinus concerning the imaging is not assessed. Nevertheless, these findings are non-specific, can vary and should be viewed in the context of the whole clinical symptomatology [3]. In odontogenic sinusitis, usually the process spreads from the maxillary sinus to the anterior ethmoids and frontal sinus [16, 20-22]. The crucial place for treatment (opening) is the ostiomeatal unit. Crovetto-Martínez et al. [20] stated that ethmoid involvement does not worsen the FESS surgical results applied to odontogenic sinusitis. Safadi et al. [22] recommend performing middle meatal antrostomy (MMA) only in any extent of PNS impairment. We perform and support the need of opening all diseased sinuses during FESS; nevertheless, in the light of Safadi et al. and Ungar et al.’s studies [21, 22], frontal sinusotomy might be a matter for ongoing discussion.

**Odontogenic sinusitis or rather SCDDT in relation to dental implants.**

According to Park [27], implants extruding more than 4 mm into the maxillary sinus, peri-implantitis and disrupted-extruded bone grafts show a significant association with implant-related sinusitis. In our view, there are five major dental implant pathologies that need to be distinguished in relation to SCDDT with sinusitis treatment management: (1) peri-implantitis without/with sinus lift, (2) implant displacement to the sinus without/with oroantral communication (fistula) or implant loss with oroantral communication (fistula), (3) implant protrusion into the maxillary sinus, (4) infected sinus lift augmentation material in the maxillary sinus, but not displaced into the sinus, (5) infected augmentation material displaced into the maxillary sinus following sinus lift. In peri-implantitis without/with SL and in implant displacement to the sinus or implant loss with OAC (OAF), the combined surgical approach is a method of choice [30]. In implant displacement to the sinus without OAC(OAF), FESS with endoscopic removal of the implant is a method of choice [30]. In implant protrusion into the maxillary sinus, FESS is recommended [9, 34]; in the case of a stable long implant with no severe peri-implantitis concurrently, implant “apicoectomy” can be considered [26]. In infected not-displaced augmentation material following SL, FESS is recommended, with a chance of implant salvage [35].

**references**

1. Fokkens WJ, Lund VJ, Hopkins C, et al. (2020) European Position Paper on Rhinosinusitis and Nasal Polyps 2020. Rhinology 58(Suppl S29):1-464. doi:10.4193/Rhin20.600
2. Felisati G, Chiapasco M. (2015) Sinonasal Complications of Dental Disease and Treatment: Prevention – Diagnosis – Management. Thieme, New York
3. Craig JR, Poetker DM, Aksoy U, et al. (2021) Diagnosing odontogenic sinusitis: An international multidisciplinary consensus statement. Int Forum Allergy Rhinol 11(8):1235-1248. doi:10.1002/alr.22777

# Misch C. (2014) Dental Implant Prosthetics. Mosby, USA

1. Stammberger H. (1991) Functional Endoscopic Sinus Surgery: The Messerklinger Technique. Mosby, USA

# Baart J, Brand H. (2017) Local Anaesthesia in Dentistry. Springer, USA

1. Costa F, Emanuelli E, Franz L, Tel A, Sembronio S, Robiony M. (2019) Fungus ball of the maxillary sinus: Retrospective study of 48 patients and review of the literature. Am J Otolaryngol 40(5):700-704. doi:10.1016/j.amjoto.2019.06.006
2. Kwon MS, Lee BS, Choi BJ, et al. (2020) Closure of oroantral fistula: a review of local flap techniques. J Korean Assoc Oral Maxillofac Surg 46(1):58-65. doi:10.5125/jkaoms.2020.46.1.58
3. Craig JR, Tataryn RW, Aghaloo TL, et al. (2020) Management of odontogenic sinusitis: multidisciplinary consensus statement. Int Forum Allergy Rhinol 10(7):901-912. doi:10.1002/alr.22598
4. Melén I, Lindahl L, Andréasson L, Rundcrantz H. (1986) Chronic maxillary sinusitis. Definition, diagnosis and relation to dental infections and nasal polyposis. Acta Otolaryngol 101(3-4):320-327. doi:10.3109/00016488609132845
5. Craig JR, Tataryn RW, Cha BY, et al. (2021) Diagnosing odontogenic sinusitis of endodontic origin: A multidisciplinary literature review. Am J Otolaryngol 42(3):102925. doi:10.1016/j.amjoto.2021.102925
6. Craig JR, Cheema AJ, Dunn RT, Vemuri S, Peterson EL. (2022) Extrasinus complications from odontogenic sinusitis: A systematic review. Otolaryngol Head Neck Surg 166(4):623-632.
7. Pokorny A, Tataryn R. (2013) Clinical and radiologic findings in a case series of maxillary sinusitis of dental origin. Int Forum Allergy Rhinol 3(12):973-979. doi:10.1002/alr.21212
8. Matsumoto Y, Ikeda T, Yokoi H, Kohno N. (2015) Association between odontogenic infections and unilateral sinus opacification. Auris Nasus Larynx 42(4):288-293. doi:10.1016/j.anl.2014.12.006
9. Vestin Fredriksson M, Öhman A, Flygare L, Tano K. (2017) When maxillary sinusitis does not heal: Findings on CBCT scans of the sinuses with a particular focus on the occurrence of odontogenic causes of maxillary sinusitis. Laryngoscope Investig Otolaryngol 2(6):442-446. doi:10.1002/lio2.130
10. Turfe Z, Ahmad A, Peterson EI, Craig JR. (2019) Odontogenic sinusitis is a common cause of unilateral sinus disease with maxillary sinus opacification. Int Forum Allergy Rhinol 9(12):1515-1520. doi:10.1002/alr.22434
11. Goyal VK, Ahmad A, Turfe Z, Peterson EI, Craig JR. (2021) Predicting odontogenic sinusitis in unilateral sinus disease: A prospective, multivariate analysis. Am J Rhinol Allergy 35(2):164-171. doi:10.1177/1945892420941702
12. Whyte A, Boeddinghaus R. (2019) Imaging of odontogenic sinusitis. Clin Radiol 74(7):503-516. doi:10.1016/j.crad.2019.02.012
13. Costa F, Emanuelli E, Robiony M. (2018) Incidence of maxillary sinus disease before sinus floor elevation surgery as identified by cone-beam computed tomography: A literature review. J Oral Implantol 44(2):161-166. doi:10.1563/aaid-joi-D-17-00209
14. Crovetto-Martínez R, Martin-Arregui FJ, Zabala-López-de-Maturana A, Tudela-Cabello K, Crovetto-de la Torre MA. (2014) Frequency of the odontogenic maxillary sinusitis extended to the anterior ethmoid sinus and response to surgical treatment. Med Oral Patol Oral Cir Bucal 19(4):e409-e413. Published 2014 Jul 1. doi:10.4317/medoral.19629
15. Ungar OJ, Yafit D, Kleinman S, Raiser V, Safadi A. (2018) Odontogenic sinusitis involving the frontal sinus: is middle meatal antrostomy enough?. Eur Arch Otorhinolaryngol 275(9):2291-2295. doi:10.1007/s00405-018-5076-3
16. Safadi A, Kleinman S, Oz I, et al. (2020) Questioning the justification of frontal sinusotomy for odontogenic sinusitis. J Oral Maxillofac Surg 78(5):762-770. doi:10.1016/j.joms.2019.12.025

# Cohen S, Hargreaves K. (2006) Pathways of the Pulp. Mosby, USA

1. Monje A, Wang H-L. (2022) Unfolding Peri-Implantitis: Diagnosis / Prevention / Management. Quintessence Publishing, USA
2. Abi Najm S, Malis D, El Hage M, Rahban S, Carrel JP, Bernard JP. (2013) Potential adverse events of endosseous dental implants penetrating the maxillary sinus: long-term clinical evaluation. Laryngoscope 123(12):2958-2961. doi:10.1002/lary.24189
3. Biafora M, Bertazzoni G, Trimarchi M. (2014) Maxillary sinusitis caused by dental implants extending into the maxillary sinus and the nasal cavities. J Prosthodont 23(3):227-231. doi:10.1111/jopr.12123
4. Park MJ, Park HI, Ahn KM, et al. (2023) Features of odontogenic sinusitis associated with dental implants. Laryngoscope 133(2):237-243. doi:10.1002/lary.30069
5. Yang J, Liu SY, Hossaini-Zadeh M, Pogrel MA. (2014) Brain abscess potentially secondary to odontogenic infection: case report. Oral Surg Oral Med Oral Pathol Oral Radiol 117(2):e108-e111. doi:10.1016/j.oooo.2013.08.011
6. Bumm CV, Folwaczny M. (2021) Infective endocarditis and oral health-a Narrative Review. Cardiovasc Diagn Ther 11(6):1403-1415. doi:10.21037/cdt-20-908
7. Felisati G, Chiapasco M, Lozza P, et al. (2013) Sinonasal complications resulting from dental treatment: outcome-oriented proposal of classification and surgical protocol. Am J Rhinol Allergy 27(4):e101-e106. doi:10.2500/ajra.2013.27.3936
8. Lopatin AS, Sysolyatin SP, Sysolyatin PG, Melnikov MN. (2002) Chronic maxillary sinusitis of dental origin: is external surgical approach mandatory? Laryngoscope 112(6):1056-1059. doi:10.1097/00005537-200206000-00022
9. Fusari P, Doto M, Chiapasco M. (2013) Removal of a dental implant displaced into the maxillary sinus by means of the bone lid technique. Case Rep Dent 2013:260707. doi:10.1155/2013/260707
10. Ata-Ali J, Diago-Vilalta JV, Melo M, et al. (2017) What is the frequency of anatomical variations and pathological findings in maxillary sinuses among patients subjected to maxillofacial cone beam computed tomography? A systematic review. Med Oral Patol Oral Cir Bucal 22(4):e400-e409. Published 2017 Jul 1. doi:10.4317/medoral.21456
11. Kim SJ, Park JS, Kim HT, Lee CH, Park YH, Bae JH. (2016) Clinical features and treatment outcomes of dental implant-related paranasal sinusitis: A 2-year prospective observational study. Clin Oral Implants Res 27(11):e100-e104. doi:10.1111/clr.12570
12. Jiam NT, Goldberg AN, Murr AH, Pletcher SD. (2017) Surgical treatment of chronic rhinosinusitis after sinus lift. Am J Rhinol Allergy 31(4):271-275. doi:10.2500/ajra.2017.31.4451
